# Creation and Evaluation of a Severity Classification of Hyperkyphosis and Hypolordosis for Exercise Therapy

**DOI:** 10.3390/life13061392

**Published:** 2023-06-14

**Authors:** David Kaps, Hannah L. Siebers, Ulrich Betz, Daniel Pfirrmann, Jörg Eschweiler, Frank Hildebrand, Marcel Betsch, Janine Huthwelker, Claudia Wolf, Philipp Drees, Jürgen Konradi

**Affiliations:** 1 Center for Mental Health, Hospital Stuttgart-Bad Cannstatt Hospital, 70374 Stuttgart, Germany; 2 Department of Orthopaedics, Trauma and Reconstructive Surgery, Uniklinik RWTH Aachen, 52074 Aachen, Germany; 3 Institute of Social Science, Media, and Sports, Johannes Gutenberg-University Mainz, 55128 Mainz, Germany; 4 Institute of Physical Therapy, Prevention and Rehabilitation (IPTPR), University Medical Center of the Johannes Gutenberg University Mainz, 55131 Mainz, Germany; 5 Department of Orthopedics and Trauma Surgery, University Hospital Erlangen of the University Erlangen-Nürnberg, 91054 Erlangen, Germany; 6 Department of Orthopedics and Trauma Surgery, University Medical Center of the Johannes Gutenberg University Mainz, 55131 Mainz, Germany

**Keywords:** back pain, spine, physiotherapy, spine shape, classification system, exercise therapy, grading system, sagittal plane, video rasterstereography

## Abstract

The rise in the occurrence of musculoskeletal disorders, such as thoracic hyperkyphosis (THK) or lumbar hypolordosis (LHL), is a result of demographic changes. Exercise therapy is an effective approach that can reduce related disabilities and costs. To ensure successful therapy, an individualized exercise program adapted to the severity of the disorder is expedient. Nevertheless, appropriate classification systems are scarce. This project aimed to develop and evaluate a severity classification focused on exercise therapy for patients with THK or LHL. A multilevel severity classification was developed and evaluated by means of an online survey. Reference limits of spinal shape angles were established by data from video rasterstereography of 201 healthy participants. A mean kyphosis angle of 50.03° and an average lordosis angle of 40.72° were calculated as healthy references. The strength of the multilevel classification consisting of the combination of subjective pain and objective spinal shape factors was confirmed by the survey (70% agreement). In particular, the included pain parameters were considered relevant by 78% of the experts. Even though the results of the survey provide important evidence for further analyses and optimization options of the classification system, the current version is still acceptable as therapeutic support.

## 1. Introduction

Musculoskeletal disorders (MSKDs) are associated with multiple impairments of daily life and different health consequences like falls or mental health limitations [1,2,3,4]. Included, lower back pain is the leading cause of disability worldwide [5,6]. Here, thoracic hyperkyphosis (THK) and lumbar hypolordosis (LHL) or flatback are common causes of lower back pain [7,8]. In addition, THK and LHL can be sequelae or symptoms of other underlying conditions beyond back pain. A significant increase can be seen in the number of people with these two MSKDs [8,9,10,11,12]. This ongoing development is the result of current issues, including demographic change or an inactive everyday life with one-sided strains [8,13,14,15,16,17,18]. However, exact prevalence values are not documented. Population-based estimates for the older population share assume a prevalence of between 20% and 40% for THKs [8]. For LHL, an age-standardized point prevalence of 9.4% can be assumed based on the close association with low back pain and the prevalence of 8.4% of other MSKDs [5,7,12].

Nevertheless, some measures promise to have a lasting positive influence on the course of the disorders [10,11,14,19]. One of them is therapeutic rehabilitation exercise [19,20,21,22,23]. Study results showed that specific exercise programs can reduce subjectively perceived pain [21,22]. Furthermore, the multifactorial induced quality of life (QOL) can be promoted by physical activity [21,24]. Meaningful evidence of exercise therapeutic methods that change the objectively measured curvature angle of the spine is not yet available [14,25,26]. For a successful treatment, it is thus even more important to positively modify the patient-specific accessory symptoms together with negative influencing factors [10,27,28]. Consequently, the therapist’s task is to create an individually tailored rehabilitation plan based on these corresponding characteristics [10,27,28]. However, a challenge here is the increasing demand for rehabilitation measures in combination with the number of trained therapists [18,29,30,31]. Therefore, it is crucial to find a solution that takes into account the individuality of each patient, as well as to counteract the personnel and structural weaknesses in exercise therapy.

Severity classifications are based on the aforementioned aspects [32,33,34,35]. These enable assigning patients to symptom-specific subgroups in an uncomplicated but still diagnostically comprehensive and resource-saving manner [28,33,35,36]. Therapeutic decision-making processes for optimal treatment can thus be facilitated and suitable exercises or entire exercise programs for exercise therapy can be designed [28,33,35]. However appropriate severity classifications established for spinal deformities are mainly aimed at patients with deformities centered in the coronal plane, such as those with scoliosis, and are mostly oriented toward surgical interventions [28,32,37,38]. Apart from accepted severity classifications for patients with THKs or LHLs, there is a fundamental lack of generally confirmed objective reference limits or intervals [8,10,28,39,40]. Only approximate values are present [8,10,11,38,39,40,41]. For thoracic kyphosis (TK) values, below 20° and above 60° are usually considered objectively pathological [8,11,37,38]. The limit values for a measurable physiological lumbar lordosis (LL) are described as 20° to 80° [11,37,38,40,42]. However, all these values are primarily based on radiological measurements, are broadly defined, vary between individual research projects, and mainly differentiate between physiological and pathological curvatures [8,37,38,40,41,42].

Beyond the striking spine shape, the concerned persons also show varied levels of, for example, pain during everyday movements and related lower mobility [8,11,14,43,44]. In addition, the patients have lower values in terms of social life and mental aspects compared with healthy control groups [11,14,44]. Despite these disparities, the existing classifications use the measurable spine shape as the single or central parameter [28,32]. However, such a method is not sufficient, even for exercise therapeutic rehabilitation [10,11,28]. Moreover, given largely invariable objective factors that lead to differences in physiological spine shape [8,10,28,41]. Accordingly, even in healthy persons, variations are common due to age, gender, ethnicity, or body stature [8,10,28,41]. Therefore, a more comprehensive, multilevel concept of severity classification is of considerable interest [28,32,39]. The patient’s individuality, as well as physical and psychological resources, are key factors [28,32,39,41].

The aim of this project was thus to develop and evaluate a severity classification scheme for THK and LHL patients, especially for exercise therapeutic rehabilitation. Using the methodological approach and the conception of a multilevel severity classification based on subjective pain grades and objectively measured spine shape parameters, a further aim was to obtain approval for the developed classification from experts in the context of a survey.

## 2. Materials and Methods

### 2.1. Collection and Methodical Implementation of Video Rasterstereography Data

Objectively measurable spinal shape indicators were considered as one component of the severity classification. The applied data were obtained from a framework project aimed at establishing a normative reference data pool for spine shapes [45]. The corresponding study was approved by the responsible ethics committee of the Medical Association of Rhineland-Palatinate (837.194.16) and is registered with the World Health Organization (WHO) (INT: DRKS00010834). Participants were recruited consecutively after media announcements according to clearly defined and exhaustive inclusion and exclusion criteria [45,46]. Based on these, participants should be primarily healthy, pain-free, and between the ages of 18–70 years. In addition, paresis and paresthesia, previous pelvic or spinal surgery, use of walking aids, and impaired motor control were deemed exclusion criteria. Each participant’s body mass index (BMI) should be less than 30 kg per meter squared. These features were checked with a standardized interview, as well as the International Physical Activity Questionnaire and appropriate mobility tests, with the Timed “Up & Go”, the Two-Minute Walk Test, the Back Performance Scale, and the Range of Motion Test amongst them [45,46]. Following the precise guidelines, 201 (132 females, 69 males) subjects were recruited and analyzed. All of them gave oral and written informed consent.

**Figure 1:**
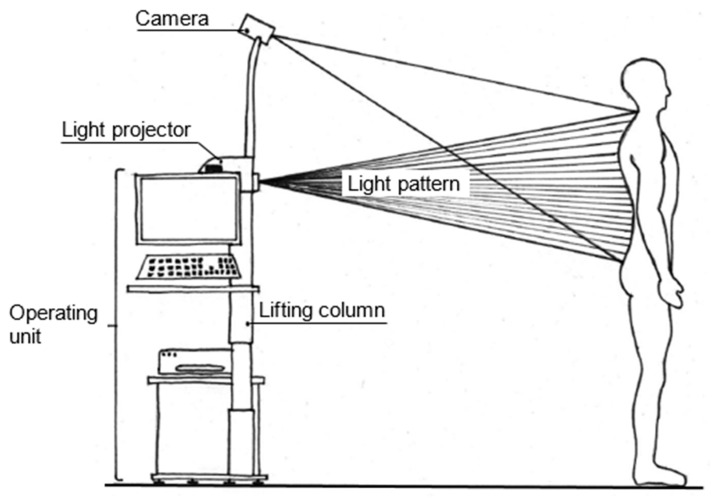
Setup of the measuring method “DIERS formetric” for the surface measurement of the video rasterstereography (based on pictures from the DIERS Company).

The established and validated DIERS formetric III 4D™, DICAM v.3.7.1.7 analyzing system (DIERS International GmbH, Schlangenbad, Germany) [47,48] was used to measure the spine (Figure 1).

**Figure 2:**
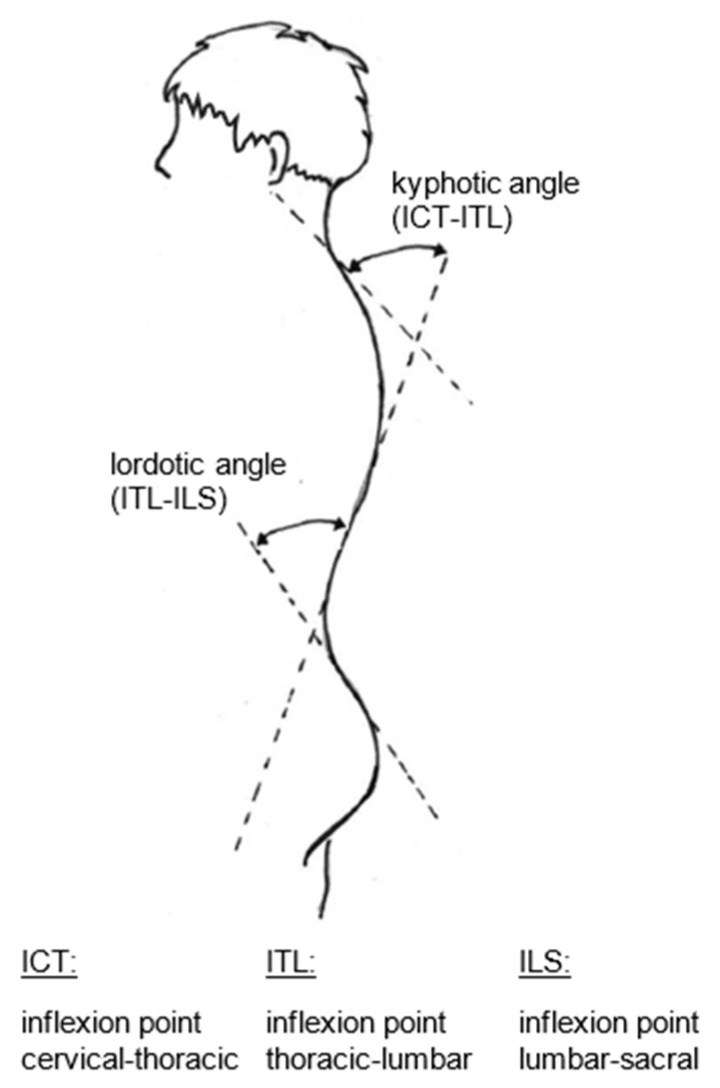
Selected angular measurements for the calculation of thoracic kyphosis and lumbar lordosis (based on pictures from the DIERS Company).

This system operates with video rasterstereography (VRS), which is based on the physical principle of triangulation [47,48]. It projects structured light onto the textile-free back of the test person (Figure 1). The inserted camera unit records the reflected light with a frequency of 60 Hz. The related software analyzes the surface topography using up to 150,000 measurement points (depending on body size). With this information about the surface curvature of the back and the anatomical landmarks identified independently at the same time, a 3D model of the spine was constructed. The model included the corresponding 3D position data for each segment of the spine, starting at the spinous process of C7 and ending at the pelvis. Corresponding parameters to describe the spine were automatically calculated and presented [45,46,47,48,49]. To further increase the measurement validity, distinctive anatomical landmarks of the back (e.g., C7, the left and right posterior superior iliac spine (PSIS), and the spine shape approximately at the level of T3 and T12) were additionally marked with infrared reflective stickers for detection by the formetric system. After the data acquisition, all measurements were visually inspected by two experienced researchers. The calculations of all TK and LL values were performed by considering fixed surface tangents (Figure 2). The TK was calculated from the angle of the tangents of ICT (inflexion point cervical-thoracic) and ITL (inflexion point thoracic-lumbar). The angle of the tangents of ITL and ILS (inflexion point lumbar-sacral) gives the degree of LL. Twelve frames were recorded for each stance measurement. Thus, twelve values were available per subject for each spine parameter [46].

The associated twelve individual values of each participant were averaged to obtain a mean parameter for each subject. Statistical figures calculated from these curvature angles were applied to establish the reference intervals. The approach utilized for this purpose by using means and their standard deviations (SDs) or percentiles is a common method [39,40,41]. Accordingly, individuals with TK or LL between the mean and ±1 SD were assigned as normative healthy references [40,41]. All values between ±1 and ±2 SDs indicated an increased sagittal curvature. Corresponding angles above or below ±2 SDs were classified as THK or LHL.

### 2.2. Evaluation of the Severity Classification

Due to various advantages, an online questionnaire was selected as the central measuring instrument for evaluating the severity classification [50]. It was developed using the open-source survey software “LimeSurvey” by LimeSurvey GmbH [51]. Expert therapists were selected as addressees. Initially, the decisive inclusion criteria were their qualifications and the field of activity. At the beginning of the survey, each participant was explicitly informed that their data would be treated anonymously and that the survey was voluntary.

Since the evaluation of the severity classification was a sub-project to evaluate a rehabilitation product, the complete questionnaire contained 35 questions. Of these, only nine were related to the current research topic and 12 to socio-demographic content. The assessment was performed using unipolar, discretely graded verbal rating scales with positive item polarity. Additional free text fields permitted personal additions.

After a pretest, the survey started on 12 December 2019 and ended on 2 February 2020.

### 2.3. Data Processing and Statistical Analysis

Before all statistical steps, the corresponding data of the VRS measurements (TK and LL) and the survey were anonymized. All following statistical procedures were performed using IBM^®^ SPSS Statistics software (version 23, v. 25, IBM Cooperation, Chicago, IL, USA). The presence of a normal distribution was determined based on the Shapiro–Wilk test with a level of significance of *α* = 0.05.

Indicators and graphs of descriptive and exploratory statistics were used when working with the VRS data and the survey findings. Location and dispersion, as well as frequency and distribution of the data, could thus be examined.

In the case of the VRS data, these characteristics were used to establish the reference limits and finally elaborate the severity classification via the outlined methodology.

When analyzing the survey results, initially, individual participants outside the inclusion criteria were ruled out. Only partially completed questionnaires without research-relevant information were excluded from all further analyses. In addition, the participants involved were divided into three expert groups (“moderately experienced experts”, “experienced experts”, or “very experienced experts”) and two age groups (≤37 years and ≥38 years) for supplementary investigations. The two age groups were recommended due to the bimodal distribution of ages. Appropriate group comparisons were examined using descriptive methods. The severity-specific answers were coded numerically. Unanswered questions were marked as missing. The highest degree of agreement always received a value of “1”, while the strongest rejection always received a value of “4”. The non-contextual statement “I cannot judge that” was coded as the number “5”. In evaluations focusing on expert groups or age groups, the respective non-context-based answer options were removed for better comparability.

To precisely determine the mean participant rating, the more meaningful arithmetic mean ($\overset{-}{x}$) was recommended instead of the median given the limited number of response categories of the rating scales. For these calculations, the 5-coded non-context response option was previously defined as a missing value. Therefore, means between one and four were possible. The interpretation of these values was consequently determined by the coding used. Irrespective of this, all figures were rounded. The qualitative results of the free text fields were examined individually and compiled in a separate file (Table A8).

## 3. Results

### 3.1. Evaluation of the Video Rasterstereographic Reference Data

Within the scope of the statistical tests, two average TK angles and one average LL angle could be identified as outliers via boxplots. The concerning subjects and their mean values were removed from the data set in order not to falsify subsequent analyses. The histograms and the Shapiro–Wilk test (TK: *p* = 0.747, LL: *p* = 0.659) confirmed a normal distribution of the measured values. The statistical analysis included 199 kyphosis angles and 200 lordosis angles. The average kyphosis angle was 50.03° (standard error: 0.58°). The 95% confidence interval of the mean value ranged from 48.89° to 51.17°. For the lordosis angle, the mean value was 40.72° (standard error: 0.63°). The lower limit of the 95% confidence interval of the mean value was 39.47° and the upper limit was 41.98°. Table A1 lists all associated statistical parameters for the two focused spinal angles of the included subjects.

### 3.2. Development of Severity Classification

For the severity classification, distinctive subjective and objective parameters were chosen, the VRS data (TK and LL) were analyzed, and the final score was generated.

Thus, the kyphosis or lordosis angle with the described measurement technique was selected as the first parameter for the multilevel severity classification (Figure 2).

To the objective spinal angles, a second parameter was added. Subjective pain perception in combination with pain-related impairments was found to be a suitable variable.

The Chronic Pain Grade (CPG) questionnaire was used as the measurement instrument in this regard [34]. It provides a tool that has been tested on patients similar to the target group, includes the desired pain parameters, defines established limit values, and offers adequate quality at the same time [52,53,54,55].

**Figure 3:**
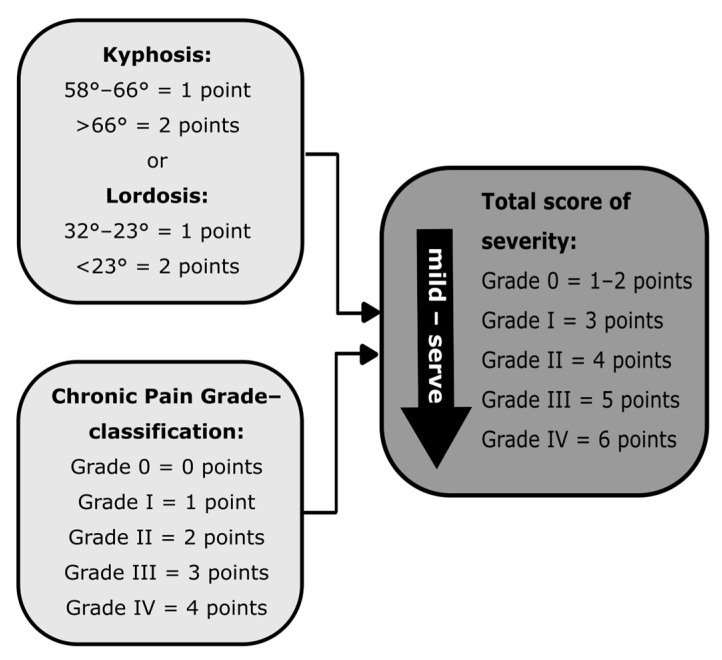
Classification system to determine the severity of thoracic hyperkyphosis or lumbar hypolordosis.

For the focused severity classification, the pain graduation of CPG and the maximum video rasterstereographic spinal angles were combined in one scheme. The extent of the disease is therefore graded using a scoring system. Both the previously defined reference intervals of the VRS data evaluation and the graduation of CPG according to Korff et al. [34] were assigned numbers for this purpose. When added up, these result in a sum between one and six. The obtained value ultimately corresponds to one of five severities, which can be taken from the classification system via the total score (Figure 3). Since initial objective proof for one of the two pathologies has to be available first, individuals with an asymptomatic TK or LL were not included in the classification system. The respective values of the interval between the mean and ±1 SD are therefore not part of the graduation.

### 3.3. Sample Description of the Online Survey

Of the 108 questionnaire returns in total, 27 female and 19 male experts commented on the severity-relevant topics, as well as two further participants without specifying their gender. Their average age was 38 (SD ± 13.47) years, whereas two individuals (4%) did not provide any details. Thus, 24 participants (50%) were classified in the younger age group (≤37) and 22 (46%) in the older group (≥38). The most represented occupational groups were physio/exercise and sports therapists (63%, *n* = 30). With 28 votes (58%), the majority of the interviewees reported that they had already been working for more than six years in exercise therapy. Based on these and other socio-demographic data, 15 persons each (31%) could be categorized as moderately experienced and experienced experts and 14 (29%) as very experienced experts. The remaining four participants (4%) could not be assigned to any expert group due to partially missing information.

### 3.4. Evaluation of the Severity Classification

The relevance of the spine shape as part of a classification system for patients with THK or LHL to choose the appropriate exercise therapeutic measure received lower ratings than the pain parameters (Table 1). The mean evaluation of the curvature angle ($\overset{-}{x}$: 1.98, SD: 0.80, *n* = 40) and the pain parameters ($\overset{-}{x}$: 1.35, SD: 0.70, *n* = 40) demonstrate this.

Results regarding the use of a severity classification that does not apply only one parameter to develop exercise therapeutic rehabilitation programs for patients with THK or LHL ($\overset{-}{x}$: 2.00, SD: 0.98, *n* = 32), the differentiation of five severity degrees in each case (THKs: $\overset{-}{x}$: 1.96, SD: 0.98, *n* = 25 and LHLs: $\overset{-}{x}:$ 1.92, SD: 0.95, *n* = 25), and the developed severity classification ($\overset{-}{x}$: 1.72, SD: 0.92, *n* = 29) are shown in Table 2.

In an additional answer-linked follow-up question to an alternative severity number for patients with THK, one participant recommended one degree of severity, another recommended more than five severities, and yet another person could not assess it. Seven participants (47%) advised a two-to-four-level concept. On the other hand, five respondents (33%) favored a completely different type of classification. In the scheme for LHLs, the feedback was almost identical. Only a single vote less was cast, meaning that the number of one degree of severity was not supported. The answers to the multiple-choice follow-up question on the developed severity classification according to alternative or supplementary severity components are shown in Table 3.

To the classification components previously covered, five experts added “ADL” (activities of daily living), impairment of respiration and dynamics of the internal organs, spinal mobility, compliance, and the ability to perform active posture correction as potential factors in an optional free text field.

Among the age groups, it was particularly evident that older survey participants were strikingly more likely to agree completely with both a multidimensional severity classification in general for determining the intensity of exertion and the designed classification. Categorized by expert status, it was noticeable that the very experienced experts most often agreed completely with the developed severity classification. Further detailed group characteristics can be found in Appendix B Table A2, Table A3, Table A4, Table A5, Table A6 and Table A7.

In turn, Table A8 documents the comments of the cross-topic free text field of the last survey page. These largely focus on the individuality of each patient and the problem of capturing this uniqueness in a general scheme.

## 4. Discussion

While research and treatment of scoliotic spinal deformities are well advanced, there is conspicuously less accepted knowledge about THKs and LHLs [8,10,32,40,56]. However, current trends, living conditions, behavioral patterns, or even social processes require effective and economic measures to be able to help affected persons appropriately [8,15,16,17,18,29,30,31]. In order to support not only the patients but also the practitioners, and to close research gaps, it was possible to develop and evaluate an appropriate severity classification. Based on a normative reference VRS dataset of the spine with the resulting objective curvature angles, combined with subjective pain parameters, a suitable scheme for the exercise therapeutic rehabilitation of patients with THK or LHL was established. This scheme takes into account both the individual symptoms and various complaints, as well as an adequate application of physical and psychological resources.

As research has already shown and the survey was able to confirm, the composition of the fitting variables was a central challenge. On the one hand, objective easy-to-measure parameters are of interest, while on the other hand, comparable angles of spine shape differ fundamentally in terms of impairment or treatment [10,28]. Accordingly, patients with an objective deformity can remain asymptomatic, while other patients with similar spinal column findings show clear adverse health consequences [10,28]. Particularly in adult patients, further disease characteristics besides the objective curvature are decisive due to the unique physiology of the spine [10,11,28]. Measuring a complex set of objective parameters would provide the desired comprehensive health status. However, this would be time-consuming and therefore less effective. Ames et al. [11] also supported a more individualized view of the patient, not least in order to incorporate disease-specific measures and personal preferences. Long-term adherence and finally a successful therapy are important aspects that consequently benefit from this [57,58,59]. The focus was hence on a combination of two parameters. On the one side, the non-invasively measured maximum curvature angles as the objective parameters. On the other side, the pain parameter is included in the assessment of the individual disability. Pain perception is an appropriate component for several reasons: the relationship between pain and spinal deformities has been well studied, pain can comprehensively describe an individual’s health status, and pain perception represents a personal state of mind that cannot be determined by one or more additional objective parameters [10,11,28,32,44,60,61]. For the pain parameter, pain-related impairments were included in conjunction with subjectively perceived pain since pain should not be measured solely based on its intensity [61]. The CPG questionnaire chosen for this purpose also enables recording pain persistence as a further strength [34]. Combined with the spinal angle, it is consequently possible to assess the broad scope of the deformity in a precise and comprehensive, yet time- and cost-saving, manner [10,11,32,61]. Simultaneously, patient-friendly handling is ensured [10,32].

Moreover, concerning the documented limited or unclear effectiveness of exercise therapy methods exclusively based on the spine shape, the integration of pain components presents a supplementary advantage [14,25,26]. Consequently, if the severity classification were based purely on the measurable curvature, the severity of a patient would change little or not at all over time. A rehabilitation program oriented toward the severities would therefore not vary either. The one-sidedness thus caused and the therapy-impairing lack of motivation encouraging low adherence would be disadvantageous consequences [57,58,59]. However, with the augmented pain variables, a diversified, severity-based treatment is possible, counteracting such negative effects.

The majority of the participating experts in the online survey also considered a severity classification consisting of several components to be relevant for the rehabilitative treatment of THKs and LHLs. The specifically designed scheme was also considered to be effective. Even those respondents who were even more critical in their initial separate assessment of the curvature angle were largely convinced by the combination with the pain parameters of the designed multilevel severity classification. The significance of the components utilized was additionally confirmed by the answers to the multiple-choice follow-up question on the developed severity classification according to alternative or supplementary severity elements (Table 3).

As the survey results indicate, the selected variables received the most support, but age and BMI were also considered significant. In addition to age and BMI, in the literature, gender; mental abilities; the occurrence of neurological symptoms; osteoporosis; and physical functions, such as spinal mobility, were explicitly highlighted [10,15,28,32,62,63]. To influence self-confidence, treatment motivation, and further psychological issues positively, aspects that do not follow a purely deficit-oriented methodology should be used, which involves reflecting the patient’s strengths, including the performance state to be recorded [58,59]. However, the inclusion of all possible variables without exception would be too extensive and would result in an extremely complex and elaborate classification. Nevertheless, it is also still unclear which criteria should be applied to effectively complete the variables used [10,32,62]. The lack of a distinct scientific consensus on the classification of patients with THK or LHL to design a generally accepted severity classification contributes significantly to this problem. Additionally, in the last free text field, the survey participants discussed the complexity of generalizing individual symptoms in a classification system (Table A8). Regardless of the current ambiguities and challenges, it will be crucial, especially for exercise therapy, to regularly determine the severity to assess the current condition and to adjust the exercises (Table A8).

Apart from the essential parameters, no binding scheme could be defined based on the survey results regarding the exact number of severities. Furthermore, the findings demonstrated the optimizable state of research concerning multilevel concepts compared with the more widespread allocations with explicitly fewer gradations. This was primarily demonstrated by the relatively high number of non-judgeable answers. According to the statements on an alternative severity number; however, it can be concluded that classification into only physiological and pathological TKs or LLs is inadequate. Naresh-Babu et al. [28] arrived at a similar conclusion. They considered several clinical–radiological subgroups in spinal deformities as being crucial [28]. On the other hand, more than five severities were also rejected by the survey participants. A differentiation of physiological and two to five pathological spinal column characteristics can thus be seen as the most practical. The defined premise for the presence of one of the two deformities and hence for grading by the classification system initially via an objectively pathological diagnosis is, however, unambiguous [10,11,32]. The physiological interval marked in this context between the mean and ±1 SD is also documented [40,41].

The significance of the results should also be judged regarding the sufficiently reliable and valid VRS technology used [47,48]. The general applicability was shown [47,48]. However, there are also results that show deviations that still need to be considered, especially for the measurement of LL [64]. X-rays in conjunction with the modified Cobb technique remain the gold standard in the diagnostic imaging of sagittal spinal deformities [13,65]. Although the innovative VRS method is thus not generally established, it offers further advantages in addition to the abovementioned limitations and strengths [47,48,66,67]. Due to the three-dimensional, non-invasive, high-resolution, and radiation- and contact-free approach, which can be carried out quickly, the procedure will be a common part of diagnostics in the future [48,66]. This is especially true for the follow-up examinations that are common for many patients with spinal deformities [66,67]. The resulting widespread usage will require corresponding classifications, such as the elaborated severity classification. Simultaneously, the existing scheme can be easily adapted for radiological-based treatments utilizing data from an appropriate reference population. Major facilitation with this process and further topics involve the acceptable correlation of the VRS angle with the angle according to Cobb [66]. Thus, fundamental transfers and comparisons are a priori possible. The decisive strengths of this research can be seen in the initial individual consideration of the patients through the combination of objective and subjective parameters, the advanced VRS technology, and the methodically structured online questionnaire.

However, the limitations of this study should be kept in mind since the number of respondents could have produced representativeness issues. Although the precisely defined target group of experts and the totality of the responses permitted an adequate interpretation of the results, the absolute number of data also had an impact on the further subdivision into age and expert groups. Thus, it was possible to identify divergent viewpoints for certain subgroups on some questions (Table A2, Table A3, Table A4, Table A5, Table A6 and Table A7), but it was not possible to identify clear opinion tendencies that were constant across all questions due to this state of affairs. It is also important to be aware of the partially unclear reasons that led to the selection of the non-context response category. Less a limitation than a future field of research arose from the values of symptom-free reference individuals used for severity classification. Even if it is normal to select healthy reference individuals, the reference intervals of the severity classification based on them should be assessed. Furthermore, concerning the reference individuals, the ratio between male (*n* = 69) and female (*n* = 132) subjects should be noted. As was previously shown, gender has an influence on the spinal anatomy [15,28]. Thus, the unequal distribution may have affected the statistical parameters evaluated. Together with the complementary variables just mentioned, as well as the further optimization and adaptation possibilities considered, practical studies on patients will be required. Thus, the applicability can be tested, and a need-based and long-term successful rehabilitation can be ensured not only for people with THK or LHL but also for various other MSKDs. In addition, the next crucial step will be to connect the different severities with appropriate rehabilitative exercise recommendations [28]. The supplementary concept designed for this purpose and the results, which were equally recorded with the survey, were found to be a suitable basis for continuing research projects. Independently of these findings, a general issue needs to be faced. Accordingly, it is advisable to conduct more multidisciplinary research on spinal deformities in the sagittal plane, aside from scoliosis, which has been the focus of the scientific literature. If the corresponding knowledge gaps are closed, the insights gained can additionally be used to optimize the current severity classification into an accepted classification system.

## 5. Conclusions

During the course of this research, it was possible to establish reference limits for TK and LL using the latest measurement technology via VRS data. With these, an individual-related scheme could be developed for patients with THK or LHL, which makes it possible to determine the severity of the respective disease for exercise therapeutic rehabilitation by means of objective spine shape angles and subjective pain parameters. The subsequent online evaluation demonstrated that the classification system has the potential to save time and costs, relieve therapists in exercise therapy, counteract personnel shortages, and guarantee rehabilitation patients a therapy that can be implemented flexibly.

However, a holistically sophisticated system, which includes all morphological body structures, as well as any personal circumstances and needs of the patients, is not feasible with the current scientific state of the art. This severity classification and its future optimizations will, nevertheless, be able to provide a basic therapy-supporting guideline for the decision-making processes of therapists and physicians on the way to the optimal treatment.

## Figures and Tables

**Table 1:** Rating of the components of the severity classification.

Severity Component	Rating				
	Very Relevant	Predominantly Relevant	Less Relevant	Not Relevant at All	Not Assessable
Angle of curvature	28% (13)	32% (15)	26% (12)	-	15% (7)
Pain components	63% (30)	15% (7)	4% (2)	2% (1)	17% (8)

Figures in brackets correspond to the number of participant votes.

**Table 2:** Rating of the severity classification and its related aspects.

Evaluation Topic	Rating				
	I Agree	I Predominantly Agree	I Agree Less	I Do Not Agree at All	Not Assessable
Multidimensional severity classification for exercise therapy	35% (12)	32% (11)	18% (6)	9% (3)	6% (2)
Severity number THK	29% (10)	24% (8)	15% (5)	6% (2)	26% (9)
Severity number LHL	29% (10)	26% (9)	12% (4)	6% (2)	26% (9)
Developed severity classification	44% (15)	26% (9)	9% (3)	6% (2)	15% (5)

Figures in brackets correspond to the number of participant votes.

**Table 3:** Participant feedback on additional severity classification components.

Component	Participant Comments	
	Number	Proportion of Cases
Angle of curvature	7	50%
Pain perception	9	64%
Pain-related impairments	13	93%
Age	6	43%
Sex	3	21%
Pelvic tilt	3	21%
Body mass index	6	43%

## Data Availability

The datasets generated and/or analyzed during the current study are not publicly available due these are part of other studies and contain personal values but are available from the corresponding author on reasonable request.

## Appendix A

**Table A1:** Statistical parameters of TK (ICT-ITL) and LL (ITL-ILS) of the included healthy subjects.

Statistical Parameter		Spinal Angle	
		Thoracic Kyphosis	Lumbar Lordosis
Number of included subjects		199	200
Mean value		50.03 (0.58)	40.72 (0.63)
95% confidence interval of the mean value	Lower limit	48.89	39.47
	Upper limit	51.17	41.98
5% trimmed mean		50.07	40.71
Median		50.56	40.89
Variance		66.94	80.48
Standard deviation		8.18	8.97
Minimum		29.09	18.06
Maximum		69.25	61.28
Range		40.16	43.22
Interquartile range		11.13	12.68
Skewness		−0.04 (0.17)	0.01 (0.17)
Kurtosis		−0.31 (0.34)	−0.35 (0.34)

TK: thoracic kyphosis. ICT: inflexion point cervical-thoracic. ITL: inflexion point thoracic-lumbar. LL: lumbar lordosis. ILS: inflexion point lumbar-sacral. The standard errors are given in brackets.

## Appendix B

**Table A2:** Cross-tabulation to the evaluation of pain components as a training plan element by age groups.

Age Group	Evaluation of Pain Components for Choosing Appropriate Exercise Therapeutic		
	Very Relevant	Predominantly Relevant	Less Relevant
Participants ≤ 37 years	5% (1)	-	5% (1)
Participants ≥ 38 years	28% (5)	11% (2)	-
Total	15% (6)	5% (2)	3% (1)

Figures in brackets correspond to the number of participant votes.

**Table A3:** Cross-tabulation to the evaluation of a multidimensional severity classification in general for the determination of the exercise intensity by age groups.

Age Group	Evaluation of a Multidimensional Severity Classification in General				
	I Agree	I Predominantly Agree	I Agree Less	I Do Not Agree at All	Total
Participants ≤ 37 years	27% (4)	27% (4)	40% (6)	7% (1)	100% (15)
Participants ≥ 38 years	50% (8)	44% (7)	-	6% (1)	100% (16)
Total	39% (12)	35% (11)	19% (6)	6% (2)	100% (31)

Figures in brackets correspond to the number of participant votes.

**Table A4:** Cross-tabulation to the evaluation of the developed severity classification by age groups.

Age Group	Evaluation of the Developed Severity Classification				
	I Agree	I Predominantly Agree	I Agree Less	I Do Not Agree at All	Total
Participants ≤ 37 years	43% (6)	57% (8)	-	-	100% (14)
Participants ≥ 38 years	60% (9)	7% (1)	20% (3)	13% (2)	100% (15)
Total	52% (15)	31% (9)	10% (3)	7% (2)	100% (29)

Figures in brackets correspond to the number of participant votes.

**Table A5:** Cross-tabulation to the evaluation of pain components as a training plan element by expert groups.

Expert Group	Cross-Tabulation to the Evaluation of Pain Components as a Training Plan Element by Expert Groups				
	Very Relevant	Predominantly Relevant	Less Relevant	Not Relevant at All	Total
Moderately experienced experts	83% (10)	17% (2)	-	-	100% (12)
Experienced experts	83% (10)	8.5% (1)	8.5% (1)	-	100% (12)
Very experienced experts	64% (9)	22% (3)	7% (1)	7% (1)	100% (14)
Total	76% (29)	16% (6)	5% (2)	3% (1)	100% (38)

Figures in brackets correspond to the number of participant votes.

**Table A6:** Cross-tabulation to the evaluation of a multidimensional severity classification in general for the determination of the exercise intensity by expert groups.

Expert Group	Evaluation of a Multidimensional Severity Classification in General				
	I Agree	I Predominantly Agree	I Agree Less	I Do Not Agree at All	Total
Moderately experienced experts	11% (1)	33% (3)	33% (3)	23% (2)	100% (9)
Experienced experts	60% (6)	40% (4)	-	-	100% (10)
Very experienced experts	33% (4)	33% (4)	25% (3)	9% (1)	100% (12)
Total	35% (11)	35% (11)	20% (6)	10% (3)	100% (31)

Figures in brackets correspond to the number of participant votes.

**Table A7:** Cross-tabulation to the evaluation of the developed severity classification by expert groups.

Expert Group	Evaluation of the Developed Severity Classification				
	I Agree	I Predominantly Agree	I Agree Less	I Do Not Agree at All	Total
Moderately experienced experts	43% (3)	57% (4)	-	-	100% (7)
Experienced experts	33% (3)	33% (3)	22% (2)	11% (1)	100% (9)
Very experienced experts	67% (8)	17% (2)	8% (1)	8% (1)	100% (12)
Total	50% (14)	32% (9)	11% (3)	7% (2)	100% (28)

Figures in brackets correspond to the number of participant votes.

## Appendix C

**Table A8:** Translated literal supplementary comments of the participants from the last free-text field, sorted by topics.

ID	Comment
17	Technical support in exercise therapy is highly desirable. However, clinical reasoning, the central therapeutic approach of physiotherapy, will not be technically feasible in the foreseeable future. Therefore, depending on the clinical situation, physiotherapists should continue to choose exercises and decide on exercise levels.
72	I consider the therapy for these patients to be very individual. A generally applicable evaluation is therefore difficult to formulate. But if possible, I have tried to mark something that applies to many of my patients.
107	The different patient profiles for flat backs and hyperkyphosis do not really enable me to make an overall assessment. However, I have nevertheless given statements as they would probably apply to most of my patients.
56	I believe that therapy is extremely individualized and that no patient can be placed in a grid. The condition of a patient is influenced by so many factors and every day anew. So I consider this assistant as a possible addition, but I don’t see any benefit or relief for my work. What you include in this assistant is what I do with every patient every day.
58	I found it very interesting to participate in the survey. Of course, I am in favor of a very individual approach to the patients.
109	In my opinion, a rehabilitation protocol should be discussed individually with the patient, especially to determine the intensity. Prior to this, it is necessary to inform a patient about pain per se and to adapt the rehabilitation protocol to his daily stress.
98	I believe that the individual symptoms of each patient and the complexity of the spine complicate general statements. My work with patients is based primarily on my own experience. Since most of the documented treatment recommendations tend to apply more to scoliosis, I was not sure about some questions, which is why I could not judge them.

ID: Number for anonymous identification of the participants.
